# Beyond POP-Q: A Scoping Review of Pelvic Floor Ultrasound for Anatomical Phenotyping of Anterior Compartment Prolapse

**DOI:** 10.3390/jcm15166149

**Published:** 2026-08-07

**Authors:** Anna Pitsillidi, Laura Vona, Stefano Bettocchi, Sven Schiermeier, Günter Karl Noé

**Affiliations:** 1 Department of OB/GYN, Rheinland Klinikum Neuss, Preußenstrasse 84, 41464 Neuss, Germany; guenter.noe@uni-wh.de; 2 Department of Medical and Surgical Sciences, Institute of Obstetrics and Gynecology, University of Foggia, 71122 Foggia, Italy; laura.vona@unifg.it (L.V.); stefano.bettocchi@unifg.it (S.B.); 3 Department of OB/GYN, University of Witten Herdecke, 58455 Witten, Germany; sven.schiermeier@uni-wh.de

**Keywords:** ultrasound, pelvic organ prolapse, lateral defects, anterior compartment, pelvic floor disorders

## Abstract

**Background/Objective:** Anterior compartment prolapse is commonly described as cystocele or anterior vaginal wall descent, although these terms do not identify the underlying mechanism of support failure. Similar POP-Q findings may result from different anatomical defects, including central cystocele, paravaginal support loss, apical descent, levator ani injury, hiatal ballooning, or combined abnormalities. Pelvic floor ultrasound may improve anatomical characterization, but its role in defect-specific phenotyping remains unclear. Our objective was to map the current evidence on the use of pelvic floor ultrasound for anatomical phenotyping of anterior compartment prolapse beyond POP-Q staging. **Methods:** A scoping review was conducted according to PRISMA-ScR guidelines. PubMed/MEDLINE, Scopus, and Web of Science were searched through May 2026. Studies evaluating anterior compartment prolapse using pelvic floor ultrasound were included. Non-ultrasound anatomical, clinical, and MRI studies were considered only as contextual literature and were not included in the evidence synthesis. **Results:** Twelve ultrasound-based studies were included. Most studies focused on lateral/paravaginal support abnormalities, while fewer investigated central cystocele configuration, apical-related anterior prolapse, levator-related abnormalities, and hiatal ballooning. Ultrasound techniques were heterogeneous and included transabdominal, introital, transvaginal, and translabial/transperineal approaches, frequently using three-dimensional and four-dimensional imaging. **Conclusions:** The available evidence suggests that anterior compartment prolapse represents a heterogeneous anatomical condition rather than a single entity defined by POP-Q descent alone. Pelvic floor ultrasound may complement clinical examination by providing dynamic information on different components of anterior compartment support, including cystocele configuration, levator ani integrity, and hiatal dimensions. However, the clinical interpretation of several phenotypes remains limited by heterogeneous terminology, variable imaging protocols, and the lack of standardized diagnostic criteria, particularly for paravaginal defects and the apical contribution to anterior wall descent. Future research should focus on harmonized ultrasound definitions, reproducible acquisition protocols, and prospective validation against anatomical and clinical outcomes to determine the role of ultrasound-based phenotyping in individualized management.

## 1. Introduction

Anterior compartment prolapse is among the most frequent forms of pelvic organ prolapse (POP) and remains a common indication for urogynecological evaluation and reconstructive surgery [1]. Although it is often described clinically as cystocele or anterior vaginal wall descent, these terms are descriptive rather than mechanistic. They indicate the compartment involved but do not identify the underlying pattern of support failure. This distinction is clinically relevant, as similar clinical findings may reflect different anatomical mechanisms, and failure to recognize the dominant defect may contribute to suboptimal surgical planning and recurrence [2].

The Pelvic Organ Prolapse Quantification system provides a standardized and reproducible method for describing the degree and location of vaginal wall descent. Nevertheless, POP-Q primarily quantifies surface anatomy. It describes how far the anterior vaginal wall descends, but it does not define why the descent occurs [3]. Similar POP-Q findings may result from different anatomical mechanisms, including central fascial attenuation, lateral or paravaginal support loss, apical descent, levator ani trauma, hiatal ballooning, or combined defects.

The heterogeneity of anterior compartment prolapse is particularly evident in the long-standing debate surrounding paravaginal defects. Lateral detachment of anterior vaginal wall support from the pelvic sidewall has been proposed as an important mechanism of cystocele formation, but clinical diagnosis remains inconsistent and imaging criteria are not standardized [4]. Studies comparing clinical examination with operative or imaging findings have reported limited accuracy and reproducibility for detecting paravaginal defects, highlighting the difficulty of identifying defect-specific anatomy by clinical assessment alone [5,6,7,8].

In addition to lateral support defects, apical support loss appears to play an important role in anterior vaginal wall descent. Several studies have demonstrated a close relationship between anterior and apical compartment support, suggesting that an apparent cystocele may not represent an isolated anterior wall defect [9]. Three-dimensional magnetic resonance imaging (MRI) studies further support this concept by demonstrating that anterior-predominant prolapse may involve midline widening, lateral/paravaginal displacement, and apical descent, with strong correlations between paravaginal and apical support abnormalities [10].

Pelvic floor ultrasound offers a dynamic, accessible, and non-invasive method for evaluating pelvic floor anatomy. Translabial and transperineal ultrasound can assess bladder neck mobility, bladder base descent, cystocele configuration, urethral rotation, retrovesical angle, levator avulsion, and levator hiatal dimensions. These parameters may help bridge the gap between descriptive clinical staging and mechanism-based anatomical phenotyping. However, while some ultrasound findings, such as levator avulsion and hiatal ballooning, are relatively well described, criteria for distinguishing central, lateral/paravaginal, apical, levator-related, and combined anterior compartment phenotypes remain insufficiently standardized [11,12].

Although narrative reviews have discussed the anatomy of anterior compartment prolapse and the role of pelvic floor imaging, most have focused on individual anatomical structures, specific ultrasound findings, or the relationship between imaging and surgical management [4,13,14]. To our knowledge, no previous review has systematically mapped the spectrum of ultrasound-defined anatomical phenotypes of anterior compartment prolapse or summarized how different ultrasound techniques have been applied to characterize the underlying mechanisms of support failure. Furthermore, the available literature uses heterogeneous terminology and diagnostic criteria, making it difficult to compare studies and translate imaging findings into a consistent anatomical framework for clinical practice.

Therefore, the aim of this scoping review is to map the current evidence on pelvic floor ultrasound for anatomical phenotyping of anterior compartment prolapse. Rather than evaluating the diagnostic accuracy of individual ultrasound signs, this review identifies and organizes the spectrum of ultrasound-defined anatomical phenotypes, summarizes the ultrasound techniques used to investigate each defect pattern, and highlights current gaps in terminology, diagnostic criteria, and clinical application. By providing a structured evidence map of the available literature, this review offers an overview of the ultrasound-based anatomical mechanisms underlying anterior compartment prolapse and may support a more individualized, mechanism-based assessment beyond descriptive POP-Q staging.

## 2. Materials and Methods

### 2.1. Search Strategy

This scoping review was conducted in accordance with the PRISMA-ScR guidelines to map and synthesize the available evidence regarding the anatomical phenotyping of anterior compartment prolapse using pelvic floor ultrasound. The review protocol was prospectively registered on the Open Science Framework (OSF) and is publicly available (registration https://doi.org/10.17605/OSF.IO/GY2PM).

A literature search was independently carried out by two reviewers (AP and LV) in PubMed/MEDLINE, Scopus, and Web of Science. The search covered all records available from database inception to May 2026, without restrictions regarding year of publication.

A combination of free-text terms and, where applicable, Medical Subject Headings (MeSH) was used to identify relevant studies. Search terms included “anterior compartment prolapse,” “anterior vaginal wall prolapse,” “cystocele,” “pelvic floor ultrasound,” “translabial ultrasound,” “transperineal ultrasound,” “3D ultrasound,” “4D ultrasound,” “paravaginal defect,” “cystocele type,” “apical support,” “levator avulsion,” and “hiatal ballooning.” Terms within the same conceptual domain were combined using the Boolean operator OR, while different concepts were combined using AND. Only full-text articles published in English were considered eligible.

**Figure 1:**
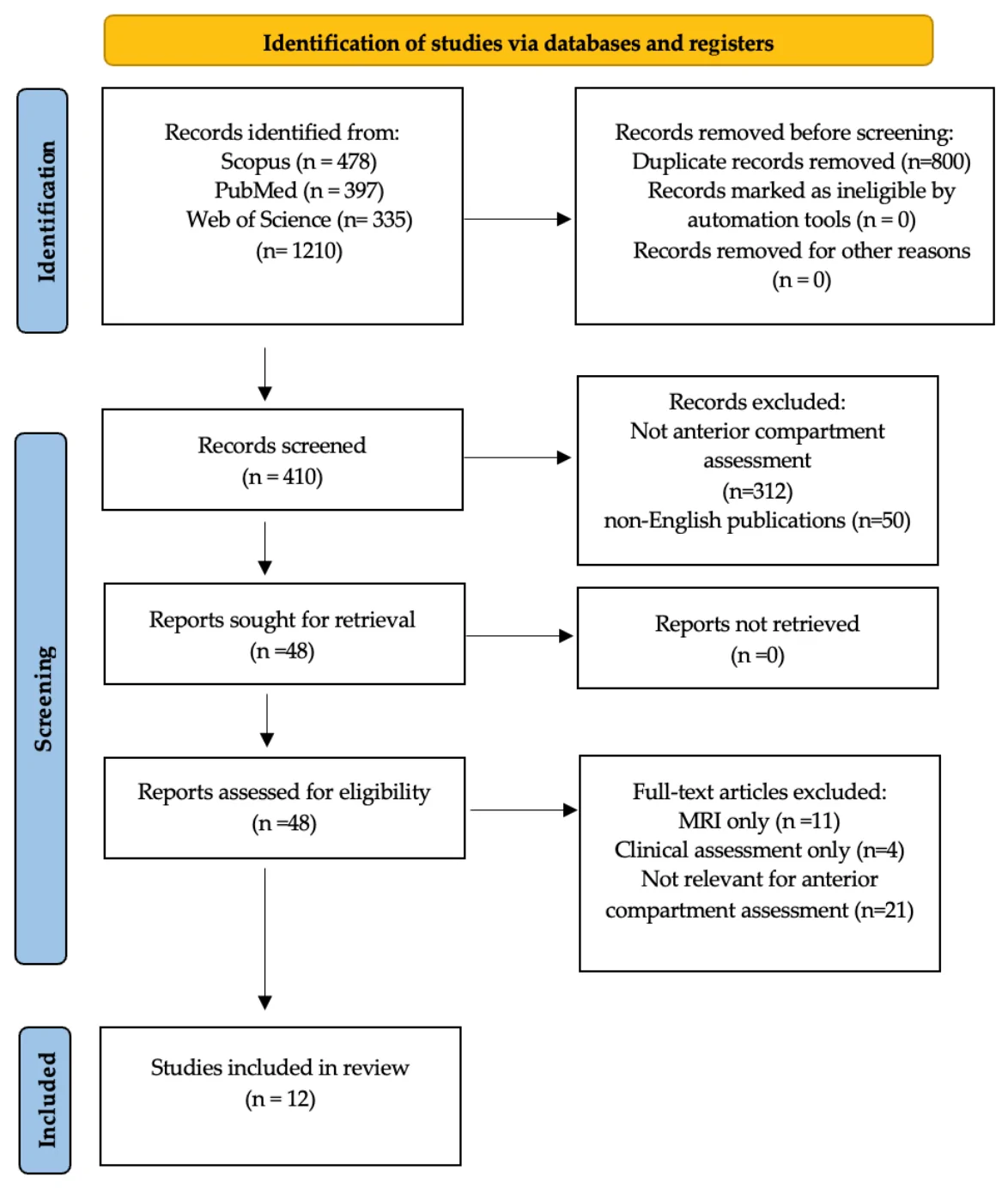
PRISMA flow chart.

The complete search strategies for PubMed/MEDLINE, Scopus, and Web of Science are provided in Supplementary Table S1. To identify additional relevant evidence, the reference lists of included studies and key review articles were manually screened. The study selection process is illustrated in the PRISMA flow chart (Figure 1) [15]. The completed PRISMA-ScR checklist is provided in the Supplementary Materials (Supplementary Table S2).

### 2.2. Eligibility Criteria

Eligible sources included original clinical, observational, diagnostic, imaging, and anatomical studies, as well as relevant narrative, systematic, or scoping reviews, provided that they evaluated pelvic floor ultrasound in relation to anterior compartment prolapse, cystocele, or anterior vaginal wall support.

The population of interest comprised women with anterior compartment prolapse, cystocele, anterior vaginal wall prolapse, pelvic organ prolapse, or pelvic floor dysfunction when anterior compartment anatomy was specifically assessed. The concept of interest was ultrasound-based anatomical phenotyping of anterior compartment prolapse, including cystocele configuration, lateral or paravaginal support abnormalities, the apical contribution to anterior wall descent, levator-related support failure, hiatal ballooning, and combined defect patterns.

Studies using translabial, transperineal, introital, transvaginal, transabdominal, two-dimensional, three-dimensional, or four-dimensional pelvic floor ultrasound were eligible.

Studies were excluded when anterior compartment assessment was performed exclusively using MRI or clinical examination without an ultrasound component. Studies focused exclusively on posterior compartment prolapse, fecal incontinence, urinary incontinence without relevant assessment of anterior compartment anatomy, or surgical outcomes without relevant ultrasound or anatomical findings were also excluded. Case reports, conference abstracts, editorials, animal studies, and non-English publications were excluded.

Non-ultrasound anatomical, clinical, and MRI studies were used only as contextual literature in the Introduction and Discussion and were not included in the formal evidence map.

### 2.3. Data Extraction and Synthesis

After removal of duplicate records, two reviewers (A.P. and L.V.) independently screened titles and abstracts according to the predefined eligibility criteria. Potentially eligible articles underwent full-text assessment, and disagreements were resolved through discussion and consensus.

Data from all included studies were independently charted by two reviewers (A.P. and L.V.) using a standardized data-charting form developed for the purposes of this review. Extracted information included study design, population characteristics, ultrasound modality, definitions of anterior compartment prolapse, anatomical phenotype classification, diagnostic criteria, and principal findings related to pelvic floor support mechanisms. Any discrepancies between reviewers were resolved through discussion and consensus. No automation tools were used for data extraction. When relevant information was unclear or incomplete in the original publications, data were recorded as reported without contacting the study authors for clarification.

When different terminology was used to describe similar anatomical concepts, studies were grouped according to the principal anatomical structure or support mechanism investigated and the corresponding ultrasound findings reported by the original authors. The resulting categories included cystocele configuration, lateral or paravaginal support abnormalities, levator ani injury, apical-related anterior prolapse, hiatal ballooning, and combined defect patterns. This grouping was used to facilitate comparison across studies with heterogeneous terminology and ultrasound approaches.

No critical appraisal or formal assessment of methodological quality was performed, as the objective of this review was to map the available evidence and summarize existing concepts rather than to evaluate study quality.

Data were synthesized descriptively through narrative analysis. Findings were organized according to anatomical phenotypes, ultrasound characteristics, and mechanisms of support failure in order to identify recurring patterns, areas of agreement, and gaps in the current literature.

## 3. Results

The database search identified a total of 1210 records, including 397 from PubMed, 478 from Scopus, and 335 from Web of Science (Figure 1). After removal of 800 duplicate records, 410 records remained for title and abstract screening. Of these, 312 were excluded because they did not address anterior compartment assessment, and 50 were excluded because they were not published in English. Consequently, 48 full-text articles were assessed for eligibility.

Following full-text evaluation, 36 studies were excluded for the following reasons: anterior compartment assessment performed exclusively with MRI (*n* = 11), studies focusing exclusively on clinical assessment without ultrasound imaging (*n* = 4), and studies not relevant to anterior compartment assessment (*n* = 21). A total of 12 studies met the predefined eligibility criteria and were included in the final evidence synthesis.

**Study characteristics**

The literature search identified 12 studies that met the predefined eligibility criteria (Figure 1). The included studies were published between 1997 and 2017 and were predominantly observational in design, including prospective observational studies (*n* = 8), retrospective observational studies (*n* = 3), and one narrative review providing anatomical and imaging background. Most studies were conducted in tertiary urogynecological settings and investigated women with pelvic organ prolapse, stress urinary incontinence, or pelvic floor dysfunction. Sample sizes ranged from 15 to 544 participants.

**Ultrasound techniques**

Most studies evaluated the anterior compartment using translabial or transperineal ultrasound, particularly three- and four-dimensional pelvic floor ultrasound. Earlier studies predominantly employed transabdominal ultrasound, whereas a smaller number used transvaginal or introital ultrasound. Ultrasound findings were compared with clinical examination in several studies, while some studies also correlated imaging findings with surgical observations or postoperative anatomical outcomes.

**Anatomical phenotypes**

The available evidence identified several anatomical phenotypes contributing to anterior compartment prolapse, including central or midline cystocele, lateral or paravaginal defects, apical-related anterior prolapse, levator ani avulsion, hiatal ballooning, and mixed defect patterns.

**Distribution of evidence**

Most studies focused on lateral/paravaginal support defects (*n* = 8), whereas fewer studies investigated central cystocele configuration (*n* = 1), apical-related anterior prolapse (*n* = 1), levator-related phenotypes (*n* = 2), and hiatal ballooning (*n* = 1). Considerable variability was observed among studies regarding the terminology used to define anatomical defects, ultrasound acquisition techniques, and diagnostic criteria. The evidence map of the included studies, including the anatomical phenotypes investigated, study designs, and ultrasound techniques used, is summarized in Table 1.

The characteristics of the included studies and the extracted data relevant to anatomical phenotyping of anterior compartment prolapse are summarized in Supplementary Table S3.

## 4. Anatomical Basis of Anterior Compartment Support

The available evidence indicates that pelvic floor ultrasound has been investigated as a complementary tool for anatomical characterization of anterior compartment prolapse; however, interpretation of the current literature requires consideration of the heterogeneity of study designs, imaging protocols, and diagnostic definitions.

The support of the anterior vaginal wall is provided by a coordinated anatomical system rather than by a single discrete fascial structure. This system includes the anterior vaginal wall and pubocervical connective tissue, their lateral attachments to the pelvic sidewall, the suspensory support of the cervix or vaginal apex, and the functional contribution of the levator ani muscle complex. DeLancey’s level theory remains a useful framework for conceptualizing these relationships: Level I support corresponds to apical suspension, Level II to lateral vaginal attachment to the arcus tendineus fascia pelvis and levator ani fascia, and Level III to distal vaginal support [9,16].

Within this anatomical framework, the pubocervical connective tissue has traditionally been considered an important element of anterior vaginal wall support, contributing to the relationship between the bladder base and the anterior vaginal wall. Attenuation or disruption of this midline support has been used to explain central or midline cystocele formation. However, anterior compartment support also depends on intact lateral attachments. The pubocervical connective tissue and anterior vaginal wall are anchored laterally toward the pelvic sidewall, particularly in relation to the arcus tendineus fascia pelvis. Loss or displacement of these lateral attachments forms the anatomical basis of the paravaginal defect concept and may result in anterior vaginal wall descent that is clinically difficult to distinguish from a central cystocele [4,16].

Apical support also contributes substantially to anterior compartment anatomy. The cervix or vaginal apex helps maintain the orientation and suspension of the anterior vaginal wall. Loss of apical support may therefore contribute to anterior wall descent, even when the leading point of prolapse appears to be in the anterior compartment. This anatomical relationship explains why cystocele and apical prolapse frequently coexist and why anterior compartment prolapse should not be evaluated in isolation [9].

Furthermore, the levator ani muscle complex provides an important functional component of anterior compartment support by maintaining the dimensions and competence of the levator hiatus. Although levator ani injury or hiatal ballooning does not represent a fascial defect of the anterior vaginal wall itself, these abnormalities may reduce the ability of the pelvic floor to counteract increases in intra-abdominal pressure. As a result, levator avulsion, impaired levator function, and excessive hiatal distensibility may contribute to anterior vaginal wall descent, prolapse progression, and postoperative recurrence risk. Therefore, levator-related abnormalities should be considered as part of a broader anatomical phenotype rather than as isolated anterior compartment defects [12,17].

Overall, anterior compartment prolapse should be regarded as the final common clinical presentation of different underlying support abnormalities rather than as a single anatomical entity. Similar degrees of anterior vaginal wall descent may result from central fascial attenuation, lateral or paravaginal displacement, apical support failure, levator-related impairment, hiatal enlargement, or combined defects. This anatomical heterogeneity provides the rationale for ultrasound-assisted phenotyping beyond descriptive POP-Q staging.

## 5. Clinical Assessment and Limitations of POP-Q

The Pelvic Organ Prolapse Quantification (POP-Q) system is the most widely used clinical tool for describing pelvic organ support. It provides a standardized and reproducible method for measuring the position of defined vaginal points relative to the hymen and has improved communication between clinicians, comparison between studies, and objective reporting of prolapse severity. In the anterior compartment, points Aa and Ba are used to describe the extent of anterior vaginal wall descent [3,18].

However, POP-Q is primarily a descriptive staging system and does not identify the underlying anatomical mechanism responsible for prolapse. A similar Ba value may reflect different patterns of support failure, including central cystocele configuration, lateral or paravaginal displacement, apical descent, levator-related impairment, or combined defects [4,10]. Therefore, while POP-Q is essential for staging and documentation, it does not provide defect-specific information that could guide individualized surgical planning.

This limitation is particularly relevant for the anterior compartment, where clinical differentiation between central and lateral support defects remains challenging. Paravaginal defects have traditionally been assessed by clinical examination, including lateral support tests with the finger or ring forceps [4]. However, studies comparing clinical assessment with operative or imaging findings have reported limited accuracy and poor reproducibility [5,7]. Similarly, interobserver and intraobserver reliability for classifying anterior vaginal wall support defects has been shown to be low [7]. These findings suggest that clinical examination alone may be insufficient for reliable defect-specific diagnosis.

In addition, anterior wall descent is often influenced by apical support. Clinical examination may identify the leading edge of the prolapse as anterior, but this does not exclude a relevant apical component [9,19]. Failure to recognize apical support loss may lead to incomplete assessment of the underlying mechanism and may contribute to recurrent anterior compartment prolapse after isolated anterior repair.

Consequently, the POP-Q system should be regarded as a necessary but incomplete tool for anterior compartment evaluation. It defines the degree of prolapse but not the anatomical phenotype. This limitation provides the basis for complementary imaging approaches, particularly pelvic floor ultrasound, which may help assess dynamic support structures and identify patterns of central, lateral, apical, levator-related, or combined support failure [8,11].

## 6. Pelvic Floor Ultrasound Techniques

Ultrasound assessment of pelvic floor disorders has been performed using multiple approaches, including transabdominal, transperineal, introital, transvaginal, and translabial imaging, often combined to improve anatomical and dynamic evaluation [20,21].

The most commonly adopted technique is transperineal ultrasound, which includes two distinct approaches: the translabial technique, performed with a convex probe placed on the labia majora or perineum, and the introital technique, in which a transvaginal probe is positioned on the perineum or immediately within the vaginal introitus [13]. Depending on equipment availability and diagnostic objectives, examinations may be performed using two-dimensional (2D), three-dimensional (3D), or four-dimensional (4D) imaging modalities.

Ultrasound examinations are generally performed with the patient in the supine lithotomy position, although some investigators have used the left lateral decubitus position [22]. Image acquisition can be obtained both at rest and during dynamic maneuvers, including maximal Valsalva and voluntary pelvic floor muscle contraction (Kegel maneuver), allowing for the assessment of pelvic organ mobility and muscular function [14].

Transabdominal suprapubic ultrasound was used to identify paravaginal defects in women with stress urinary incontinence, with examinations performed using a full bladder and repeated after voiding to assess changes in pelvic support structures [23,24]. This approach allowed visualization of pelvic support anatomy and comparison between symptomatic and asymptomatic subjects [24]. Transperineal ultrasound was used to evaluate bladder neck position and mobility through angular measurements (γ angle) relative to the symphysis pubis, assessed at rest, during Valsalva maneuver, and during pelvic floor contraction [25]. In addition, transabdominal and introital ultrasound were used to assess paravaginal defects and post-surgical anatomical changes, demonstrating the role of dynamic imaging in functional assessment [25].

Standard midsagittal imaging enables visualization of the urethra, bladder, urethrovesical junction, vaginal walls, uterus, and anorectal region, providing information on the relative position and mobility of pelvic viscera. Dynamic assessment may include measurement of the posterior urethrovesical angle and the anorectal angle both at rest and during straining or pelvic floor contraction [26]. The anorectal angle is defined by the intersection between the longitudinal axis of the anal canal and the posterior rectal wall and may serve as an indirect indicator of levator ani and puborectalis muscle activity [22]. Changes in anorectal angle displacement during contraction have also been proposed as a form of visual biofeedback reflecting pelvic floor muscle recruitment [27].

Real-time transvaginal ultrasound enabled visualization of the levator ani muscle complex and anterior vaginal structures using the symphysis pubis as an anatomical reference in the axial plane [28]. Evaluation of puborectalis muscle function can be performed by comparing measurements obtained at rest and during contraction, as the muscle normally demonstrates anterior and cranial displacement during active contraction [13].

Three-dimensional and four-dimensional ultrasound techniques provide improved visualization of the levator hiatus, pelvic floor musculature, and anal sphincter complex. Three-dimensional transperineal imaging is typically acquired with a curvilinear or transvaginal probe positioned on the perineum or labia, ensuring inclusion of both the symphysis pubis and anorectal region within the midsagittal reference plane [10]. This acquisition permits reconstruction of the axial plane used to evaluate levator hiatus morphology, dimensions, integrity, and functional changes during dynamic maneuvers [29]. More recently, 4D translabial ultrasound with tomographic acquisition was used to evaluate vaginal fornices and pelvic support defects through serial slices above the plane of minimal hiatal dimensions [30].

Beyond the assessment of pelvic organ prolapse and support defects, pelvic floor ultrasound has also been applied to the evaluation of urethral diverticula, postoperative mesh implants, and post-void residual urine volume [31,32]. Although numerous studies have investigated the use of 2D, 3D, and 4D ultrasound in women with pelvic floor symptoms, the precise role of pelvic floor ultrasonography within diagnostic pathways continues to be refined.

Basic reference landmarks included the symphysis pubis, bladder neck, bladder base, urethrovesical junction, levator ani complex, puborectalis muscle, anorectal junction, and lateral pelvic support structures such as the arcus tendineus fascia pelvis and paravaginal tissues, which were consistently used to orient imaging and define anatomical relationships [23,28].

## 7. Sonographic Parameters in Anterior Compartment Prolapse

**Bladder neck descent and mobility**

Bladder neck position and mobility were evaluated using the γ angle, defined relative to the symphysis pubis and measured at rest, during Valsalva maneuver, and during pelvic floor contraction [25]. Bladder neck descent was used as an indicator of impaired pelvic support and was assessed dynamically to characterize the degree of anterior compartment dysfunction and prolapse severity [33]. However, bladder neck descent did not consistently correlate with clinically detected paravaginal defects, suggesting limited specificity when used as an isolated sonographic marker [34].

**Bladder base descent**

Bladder base descent, including cystocele descent during straining, was assessed as a marker of anterior vaginal wall support failure. Transperineal ultrasound enables evaluation of pelvic floor and organ descent during Valsalva, facilitating the identification of prolapse across different pelvic compartments [22]. Displacement of the bladder base during straining was used to quantify prolapse severity and postpartum pelvic floor changes [33].

**Urethral rotation and urethral mobility**

Urethral mobility was assessed through angular measurements of the urethrovesical junction, particularly the γ angle and related positional changes in the bladder neck during Valsalva and pelvic floor contraction [25]. These measurements provided an indirect assessment of urethral rotation and support integrity.

**Retrovesical angle**

The retrovesical angle, formed between the proximal urethra and the bladder base, was used to evaluate urethral mobility and bladder neck support. Dynamic assessment during straining has been proposed as an indicator of anterior compartment dysfunction and urethral hypermobility [35,36].

**Levator hiatus**

Levator ani morphology was evaluated through measurements of the levator hiatus. Hiatal area was assessed using transvaginal or translabial ultrasound, and enlargement of the hiatus was consistently associated with increasing pelvic organ prolapse severity [28]. Hiatal enlargement was therefore considered a key morphological marker of pelvic floor dysfunction and loss of anterior compartment support [37].

**Levator avulsion**

Integrity of the levator ani complex, particularly the pubococcygeus/pubovisceral muscle and its lateral vaginal attachments, was assessed sonographically [28,38]. Levator avulsion was subsequently characterized using tomographic 4D ultrasound imaging and classified as partial or complete according to abnormalities observed across consecutive tomographic slices [33]. Levator ani trauma and detachment from lateral pelvic support structures were considered markers of structural pelvic floor impairment [39].

**Organ descent relative to the symphysis pubis**

Although clinical POP-Q staging was frequently used as the reference standard, correlations between sonographic measurements and POP-Q findings were variable, depending on the parameter evaluated and the imaging technique employed [28,40]. The symphysis pubis served as the principal sonographic reference landmark for evaluating pelvic organ descent. For sonographic prolapse measurements, a reference line is commonly drawn parallel to the inferoposterior margin of the symphysis pubis, allowing for standardized quantification of organ descent during maximal Valsalva [41]. Measurements of bladder neck position, bladder base descent, and related support defects were obtained relative to this fixed bony structure, allowing standardized assessment of pelvic support impairment and prolapse severity [25,33]. Differences between sonographic and clinical prolapse measurements partly reflect the use of distinct reference systems, with ultrasound relying on the symphysis pubis rather than the hymenal plane adopted in the POP-Q system.

**Paravaginal defects**

Paravaginal defects were assessed using both direct and indirect sonographic criteria. Early transabdominal suprapubic ultrasound studies focused on direct visualization of the paravaginal spaces, allowing identification of unilateral and bilateral defects as well as qualitative assessment of defect severity [24]. Defects were characterized by separation of the lateral vaginal support structures from the pelvic sidewall and showed good agreement with intraoperative findings, supporting the ability of ultrasound to depict paravaginal support loss and its surgical correction [23,25].

Subsequent approaches incorporated quantitative measurements based on lateral displacement (“sagging”) of the bladder base below the level of the centrally supported bladder base. Defect severity was expressed as the vertical distance between a reference line and the lowest point of the defect, allowing bilateral assessment of paravaginal support loss [40]. Measurements were influenced by bladder filling and vaginal balloon volume, highlighting the dynamic nature of anterior compartment support structures and the importance of standardized loading conditions during imaging [40].

Later studies using translabial, transvaginal, and tomographic 4D ultrasound shifted attention toward indirect markers of lateral support defects. Loss of vaginal or forniceal tenting, disruption of the normal vaginal “H-shape” appearance, and detachment of the anterior vaginal sulcus from its lateral supports were proposed as indicators of paravaginal injury and were associated with prolapse severity and cystocele descent [25,33,34]. In contrast, bladder base asymmetry and bladder neck mobility showed limited diagnostic value for identifying paravaginal defects when considered in isolation [34]. Ultrasound was also used postoperatively, with the disappearance of previously visualized defects after successful repair suggesting restoration of lateral vaginal support structures [25].

## 8. Ultrasound-Based Anatomical Phenotyping

Pelvic floor ultrasound may complete the clinical examination by providing a dynamic assessment of the anatomical structures involved in anterior compartment support. However, the current evidence supporting ultrasound-based anatomical phenotyping is mainly derived from observational studies, with substantial variability in ultrasound techniques, acquisition protocols, and diagnostic criteria. Therefore, the interpretation of sonographic phenotypes should be considered exploratory rather than definitive. Unlike POP-Q, ultrasound may provide information on cystocele configuration, bladder neck and bladder base mobility, levator ani integrity, and hiatal dimensions. In this context, ultrasound may support a more mechanism-based evaluation of anterior compartment prolapse. However, its ability to identify specific support defects varies between phenotypes. While cystocele configuration, levator avulsion, and hiatal ballooning are relatively well described sonographically, lateral or paravaginal defects remain less standardized and are more difficult to distinguish from apical or levator-related abnormalities [8,11].

### 8.1. Central Defect/Cystocele Configuration

Midline cystocele has traditionally been interpreted as anterior vaginal wall descent related to attenuation or weakening of midline support. From a sonographic perspective, however, this pattern is more appropriately described as cystocele configuration, because pelvic floor ultrasound characterizes the morphology and dynamics of bladder descent rather than directly visualizing a discrete pubocervical fascial defect. Ultrasound focuses on the relationship between the bladder base, bladder neck, urethra, and retrovesical angle, allowing differentiation between cystourethrocele and isolated cystocele patterns. This approach may provide clinically relevant information on anterior compartment morphology, although it does not establish whether the underlying mechanism is a true central fascial defect or part of a more complex support abnormality [10,11].

Chantarasorn and Dietz evaluated cystocele type using both clinical examination and four-dimensional pelvic floor ultrasound in 94 patients assessed with POP-Q, ultrasound, and urodynamic testing. Cystoceles were classified as cystourethrocele, corresponding to Green type II, or isolated cystocele, corresponding to Green type III. The retrovesical angle on pelvic floor ultrasound was used to differentiate cystocele type. Clinical diagnosis showed moderate interobserver agreement and moderate-to-good agreement with ultrasound findings. This supports the role of ultrasound in describing cystocele configuration and differentiating morphological subtypes of anterior compartment prolapse [11].

Nevertheless, cystocele configuration should not be interpreted as definitive evidence of a specific anatomical defect. Although ultrasound can distinguish patterns such as cystourethrocele or isolated cystocele, this classification is based on morphology rather than direct visualization of central fascial disruption [11]. Therefore, central cystocele remains a useful sonographic phenotype, but its relationship to true midline fascial defects requires further validation, particularly because anterior prolapse may involve overlapping midline, lateral/paravaginal, and apical components [10].

### 8.2. Lateral/Paravaginal Defect

Lateral or paravaginal defects have traditionally been described as loss or detachment of the lateral anterior vaginal wall support from the pelvic sidewall, particularly in relation to the arcus tendineus fascia pelvis [4]. From a phenotyping perspective, this pattern is clinically relevant because lateral support failure may present as anterior vaginal wall descent and may be difficult to distinguish from a central cystocele on routine examination. However, despite its long-standing anatomical and surgical relevance, the definition and diagnosis of paravaginal defects remain controversial. Clinical signs such as lateral sulcus descent, preservation of vaginal rugae, or reduction in the cystocele with lateral support have been proposed, but their reliability is limited [4,6].

Several studies have demonstrated that clinical assessment alone is insufficient for reliable identification of paravaginal defects. Barber et al. reported limited accuracy of preoperative clinical examination when compared with intraoperative findings [5], while Segal et al. found low sensitivity for detecting unilateral and bilateral paravaginal defects before surgery [6]. Similarly, Whiteside et al. showed poor interobserver and interobserver reliability for clinical classification of anterior vaginal wall support defects [7]. These findings highlight the difficulty of using physical examination alone for defect-specific diagnosis and surgical planning.

Ultrasound has been explored as a potential tool for identifying lateral defects. Early studies using transabdominal or introital ultrasound suggested that paravaginal defects could be visualized before surgery and that repair could be assessed postoperatively [24]. However, subsequent work raised important concerns regarding reproducibility and validity. Dietz et al. found poor agreement between clinically diagnosed lateral defects and 2D/3D translabial ultrasound findings. Although loss of vaginal tenting on Valsalva was weakly associated with clinically suspected lateral defects, the finding was not considered sufficiently reliable as a diagnostic criterion [34].

Additional limitations have been demonstrated with other ultrasound approaches. Nguyen et al. showed that sonographic paravaginal defects could be influenced by vaginal balloon volume, suggesting that some imaging findings may be artefactual rather than true anatomical defects. Thus, while ultrasound may visualize asymmetry, loss of tenting, or altered bladder base configuration, these findings cannot currently be considered validated diagnostic criteria for paravaginal defects [42].

Overall, paravaginal support failure remains one of the most clinically important but least standardized phenotypes of anterior compartment prolapse. Current evidence suggests that ultrasound may provide useful visual information on lateral support patterns, but available studies have reported sonographic signs that are indirect and may overlap with apical descent or levator-related abnormalities. Therefore, standardized imaging definitions, reproducibility studies, and validation against anatomical or surgical reference standards are needed before ultrasound-based diagnosis of paravaginal defects can be incorporated into routine clinical decision-making.

### 8.3. Apical Contribution to Anterior Wall Prolapse

Apical support is a major determinant of anterior compartment position. Although anterior vaginal wall prolapse is often clinically described as cystocele, several studies have shown that descent of the cervix or vaginal apex may contribute substantially to anterior wall descent. Therefore, an apparent anterior compartment prolapse may reflect apical support failure rather than an isolated anterior vaginal wall defect [9,19].

Summers et al. evaluated the relationship between anterior and apical compartment support and demonstrated a strong association between anterior vaginal wall descent and apical support loss [9]. Similarly, Rooney et al. reported that advanced anterior vaginal wall prolapse was highly correlated with apical prolapse, supporting the concept that the anterior and apical compartments should not be assessed independently [19]. Hsu et al. further showed on dynamic MRI that anterior compartment prolapse is related to changes in anterior vaginal wall length and support geometry, reinforcing the view that anterior wall descent is not simply a local bladder descent problem [43].

From a phenotyping perspective, these findings suggest that anterior compartment assessment should not be limited to bladder base descent or cystocele morphology. Dynamic evaluation of the cervix or vaginal apex during Valsalva is necessary to identify whether anterior wall descent occurs in isolation or as part of apical support failure. In this context, apical-related anterior prolapse represents an important mechanism-based phenotype. However, standardized ultrasound criteria for defining the apical contribution to anterior wall descent have not yet been established, and further validation is required before this concept can be routinely applied in sonographic classification.

### 8.4. Levator Ani-Related Support Failure

Levator-related abnormalities represent a distinct functional phenotype in pelvic organ prolapse. Unlike central or paravaginal defects, levator avulsion does not indicate disruption of the anterior vaginal wall or pubocervical fascia itself. Instead, it reflects injury or detachment of the levator ani muscle complex, which may reduce dynamic pelvic floor support and increase the dimensions of the levator hiatus during strain. In this way, levator trauma may contribute to anterior vaginal wall descent, prolapse progression, and recurrence risk, even though it does not define a central, lateral, or apical defect [2,17].

Several studies support the association between levator ani abnormalities and pelvic organ prolapse. DeLancey et al. showed that women with pelvic organ prolapse had more levator ani defects and impaired levator function compared with women without prolapse. Dietz and Simpson further demonstrated, using 3D/4D translabial ultrasound, that levator trauma was associated with pelvic organ prolapse [44]. In addition, Dietz et al. compared clinical palpation with rendered 3D/4D translabial ultrasound and tomographic ultrasound imaging for the diagnosis of levator avulsion, supporting the role of ultrasound as an objective tool for assessing levator integrity [45].

From an ultrasound-based phenotyping perspective, levator assessment is one of the more established components of pelvic floor imaging. Three-dimensional and four-dimensional translabial ultrasound have been used to evaluate the levator ani in the axial plane and to detect major levator defects or avulsion, although interpretation may depend on acquisition protocols and operator expertise. However, levator-related findings should be interpreted as part of the broader anatomical context of anterior compartment prolapse rather than as isolated anterior vaginal wall support defects. Their clinical value may lie mainly in risk stratification, counselling, and identification of patients with global pelvic floor support impairment.

### 8.5. Hiatal Ballooning/Levator Hiatus Enlargement

Hiatal ballooning represents excessive enlargement of the levator hiatus during Valsalva and reflects a global impairment of pelvic floor support. Unlike central or paravaginal defects, it does not describe a localized anterior vaginal wall support defect. Instead, it indicates that the pelvic floor opening through which the pelvic organs descend is enlarged, thereby reducing the ability of the pelvic floor to resist downward displacement during increases in intra-abdominal pressure [46].

Dietz et al. evaluated levator hiatal dimensions using 3D/4D translabial ultrasound in a large urogynecological cohort and demonstrated that levator hiatal area was strongly associated with prolapse symptoms and clinical signs of pelvic organ prolapse. The authors proposed a hiatal area of more than 25 cm [2] during Valsalva as a threshold for abnormal distensibility or “ballooning” [12].

In anatomical phenotyping, hiatal ballooning represents a global marker of pelvic floor distensibility rather than a compartment-specific defect. It may influence the severity and recurrence risk of anterior compartment prolapse by increasing the functional opening through which pelvic organs descend. Thus, levator hiatal assessment may complement evaluation of central, lateral, apical, and levator-related abnormalities, but should not be interpreted as a stand-alone explanation for anterior wall descent.

## 9. Clinical Implications for Surgical Planning

The clinical value of anatomical phenotyping lies in its potential to distinguish between different mechanisms of anterior compartment prolapse that may appear similar on POP-Q examination. POP-Q staging describes the degree of vaginal wall descent but does not determine whether the dominant mechanism is central, lateral/paravaginal, apical, levator-related, or combined [3]. Therefore, ultrasound-assisted phenotyping may provide additional anatomical information that complements clinical examination.

In patients with a predominantly central cystocele configuration, anterior colporrhaphy or native-tissue anterior repair may be appropriate, as these procedures primarily address midline anterior vaginal wall support [47]. In contrast, when anterior wall descent is associated with apical support loss, isolated anterior repair may be insufficient, and concomitant apical suspension may be required to restore the relationship between the apex and the anterior vaginal wall [9]. This is supported by studies demonstrating a close relationship between anterior wall descent and apical prolapse.

For suspected lateral or paravaginal defects, defect-specific paravaginal repair has been proposed to restore lateral support of the anterior vaginal wall [47]. However, the clinical application of this concept is limited by the difficulty of reliably identifying true paravaginal defects before surgery. Previous studies comparing clinical examination with operative or imaging findings have reported variable diagnostic performance, while ultrasound criteria have been investigated using heterogeneous approaches [4]. Therefore, although lateral/paravaginal phenotyping is potentially relevant for surgical planning, current evidence does not yet support routine ultrasound-based selection of patients for paravaginal repair [4].

Levator ani avulsion and hiatal ballooning have different clinical implications. These findings do not represent discrete anterior vaginal wall defects and do not directly indicate a specific anterior repair technique. Instead, they may identify patients with reduced global pelvic floor support, increased hiatal distensibility, or potentially higher risk of prolapse progression and recurrence [12,46]. Their main value may therefore lie in preoperative counselling, risk stratification, and individualized follow-up rather than in direct selection of a specific surgical procedure.

Overall, ultrasound-assisted anatomical phenotyping may support a more individualized approach to anterior compartment prolapse assessment. However, the current evidence remains insufficient to define a standardized ultrasound-based surgical algorithm. Future studies should evaluate whether preoperative ultrasound phenotyping improves procedure selection, reduces recurrence, and improves patient-reported outcomes.

## 10. Limitations

This scoping review has several limitations. The included studies were heterogeneous in terms of study design, patient population, ultrasound technique, and terminology, which limits direct comparison between studies and precluded quantitative synthesis. In addition, some of them addressed POP or pelvic floor dysfunction more broadly rather than anterior compartment prolapse specifically, so some evidence was derived indirectly. Furthermore, sonographic criteria for paravaginal defects and for the apical contribution to anterior wall descent remain insufficiently standardized. Finally, no formal risk-of-bias assessment was performed, in accordance with the scoping review design.

## 11. Conclusions and Future Perspectives

Anterior compartment prolapse should not be regarded as a single anatomical entity defined only by the degree of anterior vaginal wall descent. Similar POP-Q findings may reflect different underlying mechanisms, including central cystocele configuration, lateral or paravaginal displacement, apical support failure, levator ani injury, hiatal ballooning, or combined defects. Ultrasound represents a promising complementary method for evaluating several of these anatomical components and may therefore complement clinical examination.

Current evidence suggests that ultrasound may be particularly useful for evaluating cystocele configuration, levator avulsion, and levator hiatal dimensions. However, its ability to identify defect-specific mechanisms varies considerably. Published studies evaluating lateral or paravaginal defects and the apical contribution to anterior wall descent have used heterogeneous definitions and criteria and available sonographic signs are often indirect. Therefore, pelvic floor ultrasound should be considered an important component of the urogynecological diagnostic armamentarium, providing dynamic anatomical information that complements POP-Q and clinical examination.

Future studies should prioritize the development of standardized terminology, uniform image-acquisition protocols, and structured training pathways to improve the reproducibility of ultrasound-based assessment. Proposed phenotypes should be validated prospectively against anatomical, surgical, and clinical reference standards. Ultimately, the relevance of ultrasound-assisted phenotyping will depend on whether it can translate anatomical information into improved procedure selection, lower recurrence rates, and better patient-reported outcomes.

## Figures and Tables

**Table 1:** Evidence map of ultrasound-based anatomical phenotypes of anterior compartment prolapse.

Anatomical Phenotype	Number of Included Studies	Study Design	Ultrasound Techniques
Central cystocele configuration	1	Prospective observational study	Translabial/transperineal 3D/4D ultrasound
Lateral/paravaginal support abnormalities	8	Prospective and retrospective observational studies	Transabdominal, introital, transvaginal, and translabial/transperineal ultrasound (2D/3D/4D)
Apical-related anterior prolapse	1	Observational study	Translabial/transperineal ultrasound (2D)
Levator-related phenotype	2	Observational studies	Translabial/transperineal 3D/4D ultrasound
Hiatal ballooning	1	Observational study	Translabial/transperineal 3D/4D ultrasound

## Data Availability

The original contributions presented in the study are included in the article. Further inquiries can be directed to the corresponding author.

## Supplementary Materials

The following supporting information can be downloaded at: https://www.mdpi.com/article/10.3390/jcm15166149/s1.

## Abbreviations

The following abbreviations are used in this manuscript:

POP-Q	Pelvic Organ Prolapse Quantification
MRI	Magnetic resonance imaging
