# Supplementary material for: Beyond POP-Q: A Scoping Review of Pelvic Floor Ultrasound for Anatomical Phenotyping of Anterior Compartment Prolapse
Source: J Clin Med. 2026 Aug 7;15(16):6149. doi: 10.3390/jcm15166149 (PMC13513243; doi:10.3390/jcm15166149)
Supplement: Supplementary file 1 [file jcm-15-06149-s001.zip › Ultrasound parameter Table S3.pdf]

Phenotype / defect pattern	Author / year	Study type	Population	How was the phenotype/defect defined?	Clinical clue	Imaging / ultrasound clue	Main finding	Surgical relevance
Central defect / midline cystocele	Chantarasorn & Dietz, 2012	Clinical and ultrasound diagnostic study	94 patients assessed with POP-Q, 4D pelvic floor ultrasound and urodynamic testing	Cystocele type was classified as cystourethrocele / Green type II or isolated cystocele / Green type III	Anterior vaginal wall descent; presence of an anterior vaginal wall groove on POP-Q examination	4D pelvic floor ultrasound; retrovesical angle used to differentiate cystocele type	Clinical diagnosis showed moderate interobserver agreement and moderate-to-good agreement with ultrasound findings	Supports ultrasound as a tool for differentiating cystocele configuration, which may have functional and surgical relevance
Lateral / paravaginal defect	Arenholt et al., 2017	Narrative review	Literature on anatomy, clinical findings and imaging of paravaginal defects	Detachment/loss of lateral anterior vaginal wall support related to the pubocervical fascia and the arcus tendineus fascia pelvis	Suspected lateral sulcus defect; ring forceps test; preserved rugal folds sometimes described	Loss of vaginal tenting, sagging/drop of bladder base, vaginal or bladder asymmetry, MRI paravaginal gap	Paravaginal support contributes to anterior vaginal wall support, but its exact role in prolapse remains unclear. Physical examination is inconsistent, and US/MRI findings may overlap with levator ani defects	May be relevant for defect-specific repair or surgical planning in anterior compartment prolapse
Lateral / paravaginal defect	Dietz et al., 2004	Prospective observational study	59 women attending urogynecology clinics for bladder dysfunction, without previous prolapse or incontinence surgery	Paravaginal defect was considered a loss of lateral/paravaginal support; on ultrasound, loss of vaginal "tenting" in the axial plane was used as possible sign of paravaginal defect	Clinical assessment of lateral and central fascial defects; lateral defects were clinically reported in 24% on the left and 32% on the right	2D/3D translabial ultrasound at rest, Valsalva and levator contraction; axial plane assessment for loss of tenting; coronal plane assessment for bladder base asymmetry	Clinical examination and ultrasound did not correlate well. Loss of tenting on Valsalva was only weakly associated with clinically suspected lateral defects	Shows that ultrasound could visualize possible lateral support abnormalities, but current criteria are not reliable enough to guide paravaginal repair
Lateral / paravaginal defect	Ostrzenski & Osborne, 1998	Prospective pilot study	16 women with genuine stress urinary incontinence and 8 asymptomatic controls	Paravaginal defect was considered loss of lateral vaginal/pelvic sidewall support, clinically suspected by reduction of cystourethrocele with ring forceps and confirmed surgically in symptomatic women	Genuine stress urinary incontinence, urethral hypermobility, cystourethrocele reduced with ring forceps	Transabdominal suprapubic ultrasound with full bladder; transverse view used to identify unilateral or bilateral paravaginal defects	Transabdominal ultrasound detected paravaginal defects in all 16 symptomatic women; surgical findings reportedly confirmed the ultrasound diagnosis. Defects were also seen in 2 asymptomatic parous controls	Suggested that screening for paravaginal defects might be useful in women with stress urinary incontinence and could influence surgical repair
Lateral / paravaginal defect	Nguyen et al., 2000	Prospective imaging study	15 women with stage 4 pelvic organ prolapse	Clinically, paravaginal defect was diagnosed	Stage 4 prolapse with coexisting	Transabdominal coronal ultrasound;	Sonographic "paravaginal defects" were seen in both	Shows that ultrasound diagnosis of paravaginal

Phenotype / defect pattern	Author / year	Study type	Population	How was the phenotype/defect defined?	Clinical clue	Imaging / ultrasound clue	Main finding	Surgical relevance
			and bilateral paravaginal defects compared with 15 nulliparous controls without prolapse or paravaginal defects	when the anterior vaginal sulcus "sagged" during support of the opposite sulcus and when cystocele was reduced by lateral support. Sonographically, a defect was diagnosed when the lateral bladder base sagged below the central bladder base	clinically diagnosed bilateral paravaginal defects; ring forceps support test	paravaginal defect measured with different vaginal balloon volumes and bladder volumes	prolapse patients and nulliparous controls. The measured size of the defect was directly related to the volume of the vaginal balloon	defects may be unreliable and should not be used alone for surgical decision-making
Lateral / paravaginal defect	Martan et al., 2002	Prospective clinical imaging study	20 women with genuine stress incontinence undergoing Burch colposuspension and paravaginal defect repair	Paravaginal defect was described as separation of unilateral or bilateral attachment of the pubocervical fascia from the arcus tendineus fascia pelvis	Genuine stress incontinence with cystourethrocele; surgical indication for Burch colposuspension and paravaginal defect repair	Transabdominal ultrasound and introital ultrasound before and after surgery; bladder neck mobility also assessed transperineally	Preoperatively, transabdominal ultrasound identified unilateral or bilateral paravaginal defects in 18/20 women. Introital ultrasound showed similar findings, except in two bilateral cases classified as unilateral. After repair, no paravaginal defects were seen on follow-up ultrasound	Suggests ultrasound may be useful for confirming paravaginal defects and assessing anatomical correction after repair
Apical-related anterior prolapse	Summers et al., 2006	Imaging/clinical study	Women evaluated for pelvic organ support / anterior and apical compartment relationship	Apical contribution was defined as the relationship between anterior vaginal wall descent and descent of the cervix/vaginal apex	Anterior vaginal wall prolapse with associated cervical/apical descent	Imaging/clinical assessment of anterior and apical compartment support	Anterior compartment prolapse was strongly associated with apical support loss; apical descent may explain a substantial proportion of anterior wall descent	Supports the need to assess and treat apical support when planning anterior compartment prolapse surgery
Levator-related phenotype	Dietz et al., 2012	Retrospective diagnostic comparison study	266 women assessed in a tertiary urogynecology unit	Levator avulsion was defined as detachment/defect of the puborectalis/levator ani muscle, assessed by palpation and 3D/4D	Pelvic floor dysfunction and signs/symptoms of POP	3D/4D translabial ultrasound; rendered volumes and multislice/tomographic ultrasound imaging	Agreement between palpation and ultrasound methods was moderate. All methods were significantly associated with symptoms, clinical	Supports ultrasound assessment of levator avulsion as part of prolapse evaluation and recurrence-risk counselling

Phenotype / defect pattern	Author / year	Study type	Population	How was the phenotype/defect defined?	Clinical clue	Imaging / ultrasound clue	Main finding	Surgical relevance
				translabial ultrasound methods			signs, and ultrasound findings of POP	
Levator-related phenotype	Dietz & Simpson, 2008	Retrospective observational ultrasound study	Women attending a tertiary urogynecology unit	Levator trauma/avulsion was defined as major defect of the pubovisceral/levator ani muscle	Pelvic organ prolapse symptoms or signs	3D/4D translabial ultrasound assessment of levator trauma	Levator trauma was associated with pelvic organ prolapse, supporting levator injury as an important contributor to prolapse pathophysiology	Supports assessment of levator integrity during prolapse evaluation and may be relevant for recurrence-risk counselling
Hiatal ballooning phenotype	Dietz et al., 2008	Retrospective ultrasound study	544 women seen in a tertiary urogynecology unit	Hiatal ballooning was defined as abnormal distensibility/enlargement of the levator hiatus, especially during Valsalva	Prolapse symptoms and clinical signs of POP	3D/4D translabial pelvic floor ultrasound measurement of levator hiatal area at rest and during Valsalva	Levator hiatal area was strongly associated with prolapse symptoms and clinical signs. The authors suggested hiatal area >25 cm² on Valsalva as abnormal distensibility or "ballooning"	Supports including hiatal area/ballooning in prolapse assessment and recurrence-risk counselling
Lateral / paravaginal defect	Ostrzenski et al., 1997	Prospective observational study	30 women with SUI and clinical evidence of paravaginal defects plus 8 controls	Paravaginal defect was defined clinically by reduction of cystourethrocele with bilateral ring forceps support and sonographically by visualization of paravaginal spaces	SUI, cystourethrocele, reduction of prolapse with ring forceps support	Transabdominal contrast colposonography using a water-filled intravaginal condom; transverse suprapubic imaging of paravaginal spaces	Paravaginal defects were visualized in all selected SUI patients; 56.7% were right-sided unilateral and 43.3% bilateral. Postoperative ultrasound confirmed persistent anatomical correction after repair	Suggested that ultrasound could identify defect laterality and document restoration of lateral vaginal support after surgery
Lateral / paravaginal defect	Athanasiou et al., 2007	Prospective observational study	43 women with POP-Q stage ≥ II and 24 controls without significant prolapse	Lateral support defect was defined as detachment or loss of support of the anterior vaginal sulcus from the lateral pelvic wall, with disruption of the normal vaginal "H-shape" appearance	Pelvic organ prolapse and loss of lateral anterior vaginal wall support	Axial 2D transvaginal ultrasound assessing anterior vaginal sulcus support, levator morphology and hiatal area	Detachment of the anterior vaginal sulcus was identified in 39.5% of women with prolapse compared with 4.2% of controls ($p = 0.002$)	Supports the concept of a lateral support defect phenotype associated with prolapse and enlargement of the levator hiatus
Lateral / paravaginal defect	Cassadó-Garriga et al., 2015	Prospective longitudinal cohort study	202 nulliparous women assessed	Presumed childbirth-related fascial/paravaginal injury	Postpartum anterior compartment	4D translabial ultrasound with tomographic	Loss of tenting increased significantly after vaginal delivery and was	Supports loss of forniceal tenting as a potential imaging

Phenotype / defect pattern	Author / year	Study type	Population	How was the phenotype/defect defined?	Clinical clue	Imaging / ultrasound clue	Main finding	Surgical relevance
			antepartum; 163 reassessed postpartum	was defined by loss of forniceal tenting on tomographic ultrasound imaging	descent after vaginal delivery	assessment of forniceal tenting	independently associated with greater postpartum cystocele descent; reproducibility was moderate (κ = 0.63)	marker of anterior fascial/paravaginal injury relevant to prolapse development
