# Supplementary material for: Beyond POP-Q: A Scoping Review of Pelvic Floor Ultrasound for Anatomical Phenotyping of Anterior Compartment Prolapse
Source: J Clin Med. 2026 Aug 7;15(16):6149. doi: 10.3390/jcm15166149 (PMC13513243; doi:10.3390/jcm15166149)
Supplement: Supplementary file 1 [file jcm-15-06149-s001.zip › Table S1. Database search strategy.pdf]

Database	*Search strategy*
PubMed/MEDLINE	("anterior compartment prolapse" OR "anterior vaginal wall prolapse" OR cystocele) AND ("pelvic floor ultrasound" OR "translabial ultrasound" OR "transperineal ultrasound" OR "introital ultrasound" OR "3D ultrasound" OR "4D ultrasound" OR ultrasound OR ultrasonography)
Scopus	TITLE-ABS-KEY (("anterior compartment prolapse" OR "anterior vaginal wall prolapse" OR cystocele) AND ("pelvic floor ultrasound" OR "translabial ultrasound" OR "transperineal ultrasound" OR "introital ultrasound" OR "3D ultrasound" OR "4D ultrasound" OR ultrasound OR ultrasonography))
Web of Science	TS=(("anterior compartment prolapse" OR "anterior vaginal wall prolapse" OR cystocele) AND ("pelvic floor ultrasound" OR "translabial ultrasound" OR "transperineal ultrasound" OR "introital ultrasound" OR "3D ultrasound" OR "4D ultrasound" OR ultrasound OR ultrasonography))
